# Defect-related hysteresis in nanotube-based nano-electromechanical systems

**DOI:** 10.1186/1556-276X-6-245

**Published:** 2011-03-22

**Authors:** Leonidas Tsetseris, Sokrates T Pantelides

**Affiliations:** 1 Department of Physics, National Technical University of Athens, GR-15780 Athens, Greece; 2 Department of Physics and Astronomy,Vanderbilt University, Nashville, TN 37235, USA; 3 Department of Electrical Engineering and Computer Science,Vanderbilt University, Nashville, TN 37235, USA; 4 Oak Ridge National Laboratory, Oak Ridge, TN 37831, USA

## Abstract

The electronic properties of multi-walled carbon nanotubes (MWCNTs) depend on the positions of their walls with respect to neighboring shells. This fact can enable several applications of MWCNTs as nano-electromechanical systems (NEMS). In this article, we report the findings of a first-principles study on the stability and dynamics of point defects in double-walled carbon nanotubes (DWCNTs) and their role in the response of the host systems under inter-tube displacement. Key defect-related effects, namely, sudden energy changes and hysteresis, are identified, and their relevance to a host of MWCNT-based NEMS is highlighted. The results also demonstrate the dependence of these effects on defect clustering and chirality of DWCNT shells.

## Introduction

The presence of point defects, namely, vacancies and self-interstitials (SI), in modern carbonbased nano-materials has been the subject of intense research investigations [1-5]. These defects correspond to high-energy configurations which are not energetically favorable under thermal equilibrium. Nonetheless, they can be formed as metastable structures because of non-equilibrium conditions during growth or under irradiation. Though they are typically harmful to device performance, control over their formation can lead to defect engineering of novel structures with enhanced functionalities.

One particular class of materials where point defects are expected to play an important role are nano-electromechanical systems (NEMS) that are based on multi-walled carbon nanotubes (MWCNTs) [6-12]. The relative displacement of MWCNT shells causes variation in the overlap of electronic states associated with neighboring tubes and change MWCNT properties. This fact, combined with the ultra-low friction for inter-shell displacement in the so-called incommensurate MWCNTs, has opened the way for a host of proposals toward novel MWCNT-based NEMS, such as nano-motors [8], nano-switches [9], or GHz oscillators [10-12].

For many types of vacancy and SI configurations, structural properties resemble those described in previous extended studies [13,14] for their counterparts on single-walled carbon nanotubes (SWC-NTs). The possibility, however, of defect-induced linking of vicinal tubes is a key difference between MWCNTs and SWCNTs and gives rise to important features in the dynamics of defects in the former systems. Evidence for the role of point defects has appeared in experiments [15-18] and molecular dynamics simulations [19-24]. Distinct defect-related features that have been discussed in past computational studies are the mechanical load transfer and oscillation damping in MWCNT-based NEMS [19-24]. Recently, we discussed [25] other important effects associated with point defects in MWCNTs. In particular, we identified the atomic-scale mechanisms that induce hysteresis and energy dissipation in NEMS-related response of double-walled carbon nanotubes (DWCNTs) with zig-zag moving shells.

In this article, we report the results of *ab initio *calculations that explore additional key aspects of defect dynamics in DWCNTs. First, we address the possibility of defect clustering and its effect on NEMS-related response of the zig-zag DWCNTs. We find that formation of defect complexes is energetically favorable and limits hysteresis during inter-tube displacement of DWCNTs. Second, we analyze the stability of point defects in arm-chair DWCNTs and their response during inter-tube sliding or inter-tube rotation. We find that hysteresis is a common tract for zig-zag and arm-chair DWCNTs, but its relative importance differs for sliding and rotation in these two classes of nano-materials. Overall, the results show that the chirality of MWCNT shells and the interactions between defects are the key factors for the employment of these systems in NEMS applications.

## Method

The results presented in this article are based on the first-principles density-functional theory calculations. We used a plane wave basis with an energy cutoff of 300 eV, and ultrasoft-pseudopotentials [26] for the interactions between valence electrons and the ionic cores, as implemented in the code VASP [27]. The exchange and correlation effects were described with a local-density approximation [28] functional. Large supercells were employed to ensure convergence of total energy differences with respect to the size of the periodic simulation box. In particular, the results presented in this article for a (9,0)@(18,0) DWCNT are based on supercells with 144 (288) atoms in the inner (outer) tube. The corresponding numbers for the (6,6)@(11,11) DWCNTs are 144 and 264 for the inner and outer shells, respectively.

## Results and discussion

**Figure 1:**
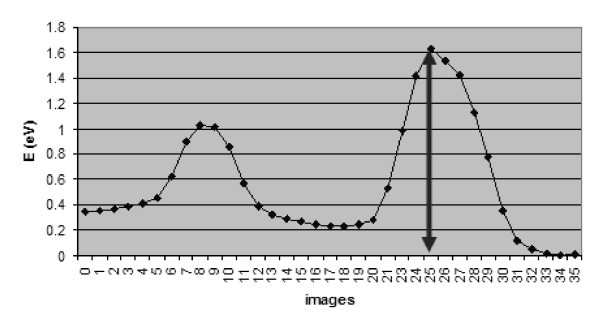
**Energy variation during consecutive migration steps of a C SI in a (9,0)@(18,0) carbon nanotube**. The arrows show the rate-limiting step that determines a diffusion barrier of about 1.6 eV.

The most stable structure [25] for a single C SI in a (9,0)@(18,0) DWCNT is an inter-tube bridge. Figure 1 shows the energy variations that accompany the successive transformations which enable this defect to migrate between neighboring sites. The results were obtained by the elastic band method [29], an approach that typically provides [30,31] barriers in satisfactory agreement with experiments. The method simulates the so-called minimum-energy pathway of a process with a sequence of intermediate configurations termed images. The curve of Figure 1 includes three local-energy minima, related to structures that play a role in the diffusion of SI through sequential switches of its bonds with the inner and outer shells. The effective activation energy for migration is about 1.6 eV.

**Figure 2:**
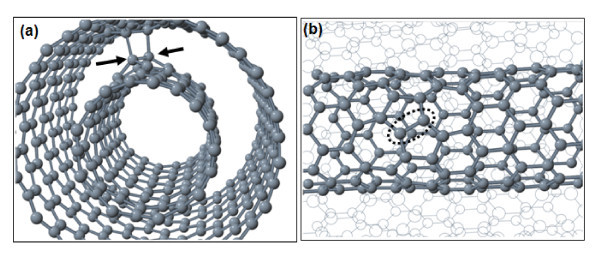
**Pairs of carbon interstitials in a (9,0)@(18,0) double-wall carbon nanotube**. **(a) **Inter-tube bridge (arrows point to C interstitials), **(b) **hillock on the inner (9,0) tube (the atoms and bonds of the outer shell are shown in a wire frame).

Given that SI defects tend to agglomerate on graphene and SWCNT [4,5], it is natural to probe whether similar trends appear for defects in DWCNTs. Indeed, pairs of SIs in a (9,0)@(18,0) DWCNT have significant binding energy against dissociation to individual defects. The most stable pair structure is shown in Figure 2b. It has a binding energy of 5.4 eV, and it resembles the hillock SI defect found on graphene and SWCNTs [4]. A large binding energy and a SI diffusion barrier of 1.6 eV suggest that the stable hillock is formed under annealing at moderate temperature above room conditions.

**Figure 3:**
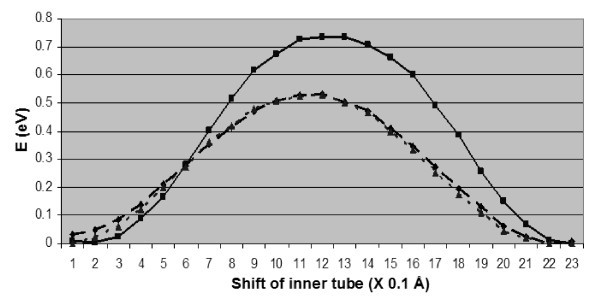
**Energy variation during inter-tube sliding of a (9,0)@(18,0) carbon nanotube with no defects (squares-solid line), with a hillock SI pair (diamonds-dashed line) or a di-vacancy (triangles-dotted line) on the inner (9,0) shell**.

Compared to the configuration of Figure 2b, the energy of a hillock structures on the outer (18,0) shell is higher by 1.35 eV, while the formation of the double inter-tube bridge of Figure 2a increases the energy by more than 1.9 eV. Clustering changes thus the character of the defect favoring the elimination of inter-shell links. As shown in Figure 3, the energy variation during sliding of the inner shell is smooth in the absence of inter-tube bridges, without the cusps that are characteristic [25] to inter-tube shift in the case of individual SI's. The presence of the hillock reduces the amplitude of the variation (also called corrugation) from 0.77 eV of the pristine case to about 0.53 eV for the case of the hillock.

As for SIs, clustering is energetically favorable also for vacancies. The lowest-energy structure is the di-vacancy on the inner (9,0) shell. The energy of a di-vacancy on the outer (18,0) tube is 0.8 eV higher, while the vacancy-induced inter-tube bridge formation increases the energy by more than 5.5 eV. The results of Figure 3 show that the presence of the di-vacancy affects the response of the (9,0)@(18,0) DWCNT under inter-tube sliding in a similar way as SI pairs. In particular, the formation of the di-vacancy does not introduce any hysteretic effects, but limits corrugation by about 0.24 eV.

**Figure 4:**
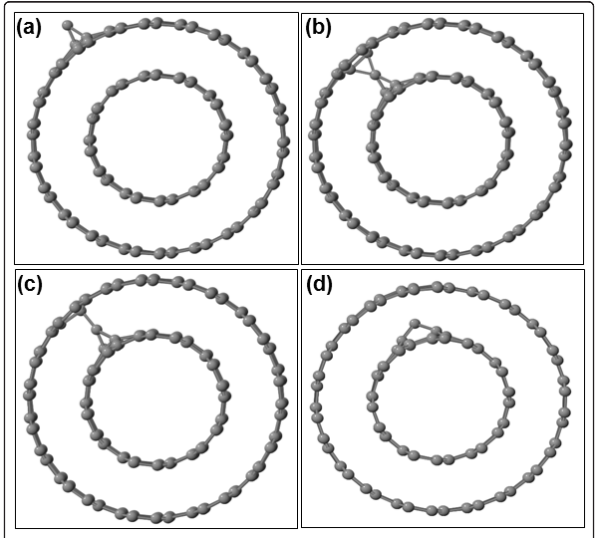
**Carbon SI in a (6,6)@(11,11) carbon nanotube: (a, d) C adatoms on the outer and inner shells, respectively, **(b, c) **inter-tube bridges**.

We now turn our attention to the stability of point defects in the arm-chair (6,6)@(11,11) DWCNT. Figure 4 depicts several SI configurations, in particular, two structures of C adatoms on the outer and inner shells, and two geometries with inter-tube SI bridges. The most stable configuration is the one depicted in Figure 4b. If we set the energy of this structure equal to zero, then the energies of the geometries of Figure 4a, c, d are higher by 1.15, 0.64, and 0.60 eV, respectively.

**Figure 5:**
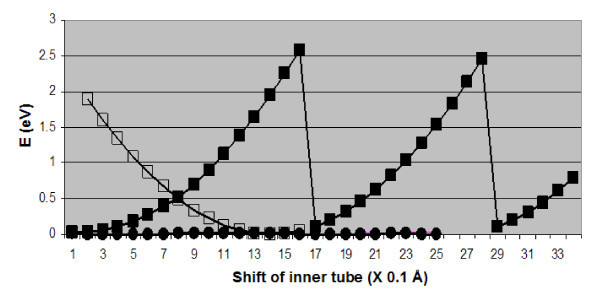
**Energy variation during inter-tube sliding of a (6,6)@(11,11) carbon nanotube with a C SI (filled and open squares for sliding in opposite directions)**. The lines with almost vanishing values (filled circles) at the bottom are for the pristine case of no defects and for a single vacancy on the inner tube.

When the inner shell is pulled with respect to the outer tube, the SI bridge is moving in the direction of sliding and the energy initially increases, as shown in the corresponding diagram of Figure 5. At a certain displacement, however, one of the bonds between the SI and the inner tube switches to a neighboring site and another cycle of stretching commences. The end result is that the SI stays roughly at the same spot during repeated cycles, despite the relative movement of the DWCNT tubes. When sliding materializes in the opposite direction, a different stretching sequence is traced, shown as open squares in Figure 5. This difference in paths gives rise to hysteresis during inter-tube sliding, highlighted by an arrow in Figure 5. Compared to the SI-related energy variation, the corresponding changes for a pristine (6,6)@(11,11) DWCNT or one with a single vacancy on the inner tube are negligible.

**Figure 6:**
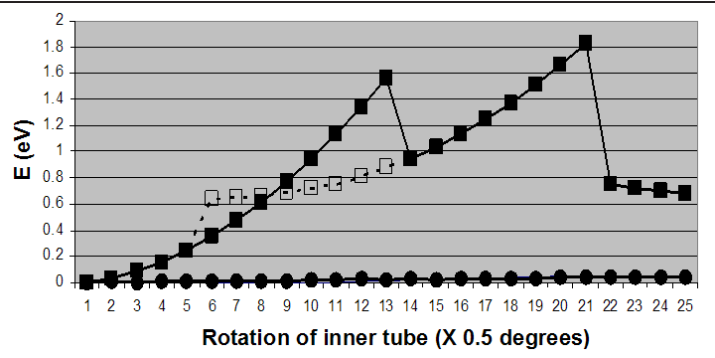
**Energy variation during inter-tube rotation of a (6,6)@(11,11) carbon nanotube with a C SI (filled and open squares for rotation in opposite angles)**. The lines with almost vanishing values (filled circles) at the bottom are for the pristine case of no defects and for a single vacancy on the inner tube.

Figure 6 shows the variation of energy when the inner shell is rotated in small angles. As in the case of sliding, the first stretching phase is suddenly interrupted through an abrupt transformation to a neighboring inter-tube configuration. Unlike sliding, however, repeated stretching cycles during rotation force the SI to move along with the inner shell, while its bonds to the outer tube switch sequentially to neighboring sites. The abrupt changes depicted in Figure 6 can give rise to hysteresis when rotation direction is reversed. This effect is shown as open squares in the figure.

## Conclusions

The energy curves of Figures 5 and 6 share some key features, namely sudden changes and hysteresis, with the corresponding diagrams for energy variation [25] during inter-tube sliding and rotation in zig-zag DWCNTs. There are, however, also important differences between the two types of nanotubes. In particular, the energy corrugation during inter-shell sliding is significant for a (9,0)@(18,0) DWCNT with no defects or with vacancies, but small in the case of the (6,6)@(111,11) DWCNT. Moreover, as we noted above, the (6,6)@(11,11) SI defect moves along with the inner-tube during rotation, but stays at the same site during sliding. The reverse is true for a (9,0)@(18,0) DWCNT defect under sliding and rotation.

Owing to the SI-related effects described above, the performance of many types of MWCNT-based NEMS may degrade in the presence of even small numbers of defects. Nevertheless, there also scenarios in which the defects can enable new features. For example, because of the sudden drops in energy during inter-tube displacement, the inter-shell bridges may be used to convert the mechanical energy supplied for sliding or rotation to thermal energy. Given that carbon nanotubes have high thermal conductivity, this excess thermal energy can be transferred to the other end of the nanotube and, thus, cause local heating of restricted spots.

In summary, we showed using the first-principles calculations that defect formation can lead to the appearance of inter-tube bridges and significant hysteretic effects in MWCNT during inter-tube displacement. The extent of these effects, however, depends strongly on the chirality of nanotube shells, and on the creation of defect complexes, which favors elimination of the inter-shell links.

## Abbreviations

DWCNT: double-walled carbon nanotube; MWCNT: multi-walled carbon nanotube; NEMS: nano-electromechanical systems; SI: self-interstitial; SWCNT: single-walled carbon nanotube.

## Competing interests

The authors declare that they have no competing interests.

## Authors' contributions

LT conceived of the study, performed the calculations and participated in the analysis and presentation of the results. STP conceived of the study and participated in the analysis and presentation of the results. All authors read and approved the final manuscript.
